# Cisplatin as a Xenobiotic Agent: Molecular Mechanisms of Actions and Clinical Applications in Oncology

**DOI:** 10.3390/jox16010009

**Published:** 2026-01-08

**Authors:** Monia Cecati, Valentina Pozzi, Veronica Pompei, Valentina Schiavoni, Stefania Fumarola, Alice Romagnoli, Giovanni Tossetta, Angelo Montana, Alessandro Polizzi, Davide Sartini, Roberto Campagna

**Affiliations:** 1 Department of Human Sciences and Promotion of the Quality of Life, San Raffaele Roma University, 00166 Rome, Italy; monia.cecati@uniroma5.it; 2 Department of Clinical Sciences, Polytechnic University of Marche, 60126 Ancona, Italy; v.pozzi@univpm.it (V.P.); v.pompei@univpm.it (V.P.); v.schiavoni@pm.univpm.it (V.S.); d.sartini@univpm.it (D.S.); 3 Advanced Technology Center for Aging Research, Istituto di Ricovero e Cura a Carattere Scientifico (IRCCS) INRCA, 60121 Ancona, Italy; s.fumarola@inrca.it; 4 Department of Life and Environmental Sciences, Polytechnic University of Marche, 60131 Ancona, Italy; a.romagnoli@staff.univpm.it; 5 Department of Biomedical Sciences and Public Health, Section of Legal Medicine, Polytechnic University of Marche, 60126 Ancona, Italy; a.montana@univpm.it; 6 Department of General Surgery and Surgical-Medical Specialties, School of Dentistry, University of Catania, 95124 Catania, Italy; alessandro.polizzi@phd.unict.it

**Keywords:** cisplatin, cis-diamminedichloroplatinum(II), chemoresistance

## Abstract

Cisplatin, a platinum-based compound, is a cornerstone of modern chemotherapy and remains widely used against a variety of solid tumors, including testicular, ovarian, lung, bladder, and head and neck cancers. Its anticancer activity is primarily attributed to the formation of DNA crosslinks, which obstruct replication and repair, ultimately leading to apoptosis. However, the clinical value of cisplatin is constrained by two major challenges: its toxic profile and the development of resistance. Cisplatin toxicity arises from its interaction not only with tumor DNA but also with proteins and nucleic acids in healthy tissues, resulting in a range of adverse effects, including, but not limited to, nephrotoxicity, ototoxicity, neurotoxicity, and gastrointestinal injury. In pediatric patients, permanent hearing loss represents a particularly debilitating complication. On the other hand, tumor cells can evade cisplatin cytotoxicity through diverse mechanisms, including reduced intracellular drug accumulation, enhanced DNA repair, detoxification by thiol-containing molecules, and alterations in apoptotic signaling. These resistance pathways severely compromise treatment outcomes and often necessitate alternative or combination strategies. This review examines the chemical structure of cisplatin, the molecular mechanisms of cisplatin cytotoxicity and cisplatin-induced resistance, as well as the main applications in cancer management and the complications associated with its clinical use.

## 1. Introduction

**Figure 1:**
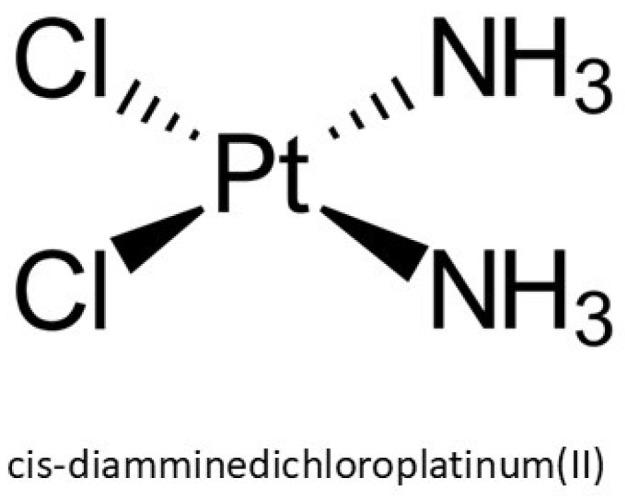
Chemical structure of cisplatin.

Over the past several decades, platinum-based chemotherapeutics, particularly cis-diamminedichloroplatinum(II), known as cisplatin (CAS No. 15663-27-1), have emerged as pivotal agents in the oncological pharmacopeia due to their robust cytotoxic efficacy against a spectrum of malignancies, including sarcomas and epithelial neoplasms. Structurally, cisplatin is a square planar coordination complex composed of a central platinum atom bonded to two chloride and two ammine ligands, with a molecular weight of 301.1 g/mol (Figure 1). Its physicochemical properties such as a high melting point (~270 °C), a density of 3.74 g/cm^3^, and aqueous solubility of 2.53 g/L at 25 °C, influence its pharmacokinetics and bioavailability [1]. Despite its chemical stability, it remains susceptible to isomerization under extended exposure, potentially diminishing its pharmacological activity. Initially synthesized by Peyrone and structurally elucidated by Werner, its therapeutic relevance was not recognized until Rosenberg’s observation of mitotic inhibition in *Escherichia coli* during the 1960s, which catalyzed its rapid adoption in clinical oncology and eventual FDA approval in the late 1970s [2]. The discovery that cisplatin was the compound mediating the observed biological response inspired extensive research into the therapeutic potential of platinum-based coordination complexes, as well as analogous compounds of palladium and other noble metals, for oncological applications.

Cisplatin’s mode of action, involving DNA crosslinking and apoptosis induction, underpins its frontline role in chemotherapeutic regimens. However, its efficacy is frequently undermined by the emergence of both intrinsic and extrinsic resistance mechanisms within tumor cells. Extrinsic resistance, often acquired through treatment-induced genomic mutations or alterations in drug target pathways, impairs cisplatin interaction with intracellular biomolecular targets. Meanwhile, intrinsic resistance, a pre-existing refractoriness, can render malignancies nonresponsive to platinum agents from the outset, posing significant hurdles to therapeutic success. Additionally, the therapeutic utility of the compound is limited by its propensity to induce dose-limiting toxicities in renal and gastrointestinal systems, necessitating combinatorial strategies to mitigate adverse effects while enhancing cytotoxic synergy. Consequently, comprehensive understanding of chemical reactivity of the cisplatin, cellular uptake, and biotransformation pathways is imperative to optimize its clinical utility and overcome resistance. As efforts continue to refine treatment protocols and develop second-generation platinum analogs, the molecular and pharmacodynamic insights into this class of drugs remain central to advancing precision oncology and overcoming the multifaceted challenges inherent to chemotherapeutic intervention.

## 2. Pharmacokinetics of Cisplatin

The conceptual breakthrough that catalyzed the incorporation of platinum-based molecules into chemotherapeutic protocols originated from experimental findings demonstrating that a cis-oriented platinum(II) coordination complex could effectively arrest the proliferation of both prokaryotic and mammalian cell lines, including aggressive tumor phenotypes in rat models [3,4]. Historically, skepticism toward the clinical use of heavy metals such as platinum stemmed from their inherent toxicity and associations with other hazardous elements like mercury. However, following extensive preclinical evaluations conducted under the supervision of the National Cancer Institute, this compound—subsequently designated as cisplatin—secured regulatory approval for oncological application by the late 1970s by the Food and Drug Administration [5]. The clinical armamentarium was later expanded to include structurally analogous agents such as carboplatin and oxaliplatin, each characterized by distinct pharmacokinetic profiles.

Unlike conventional small-molecule anticancer agents, platinum drugs exhibit distinct coordination chemistry in aqueous media. Under physiological pH (~7.2), cisplatin undergoes aquation, progressively replacing chloride ligands with hydroxyl groups, leading to the formation of dihydroxo species [6]. This reaction proceeds with a half-life of about one hour at body temperature (~35.5 °C). Once introduced intravenously, the majority of circulating cisplatin (68–98%) binds rapidly to plasma proteins, particularly albumin [7,8,9]. Each albumin molecule can coordinate multiple cisplatin units via histidine and methionine residues [6]. Renal elimination accounts for the excretion of both unbound cisplatin and a minor fraction of protein-associated complexes [10].

The cytotoxic effect of cisplatin and its analogues carboplatin and oxaliplatin is attributed to interference with nuclear DNA processes, preventing both duplication and repair, thereby halting cellular proliferation. For this effect to occur, the drug must traverse the plasma membrane and accumulate in the nucleus. Entry occurs largely through passive diffusion, with uptake proportional to extracellular drug levels up to approximately 3 mM [11]. A possible, though controversial, auxiliary mechanism involves copper transporters (SLC31A1 and SLC31A2). These proteins, containing methionine- and histidine-rich extracellular domains, could theoretically mimic albumin in binding platinum complexes [12]. Nonetheless, experimental evidence suggests that platinum–sulfur interactions in these transporters are neither stable nor favorable, raising doubts about their physiological role. At present, the contribution of copper transporters to platinum drug uptake remains uncertain [13].

Another proposed mechanism for cellular uptake of cisplatin involves members of the solute carrier 22 (SLC22A) family, which encompasses both organic cation transporters (OCTs) and organic anion transporters (OATs). The hypothesis concerning OCT-mediated import is grounded in the fact that, once dissolved in aqueous media, cisplatin undergoes hydrolytic transformations that yield positively charged complexes, which could represent potential substrates for cation transporters. This assumption gains plausibility from the observation that OCTs are abundantly expressed in organs and tissues commonly affected by cisplatin-induced toxicity [14]. In parallel, it has been argued that cisplatin, under physiological conditions, can also form carbonate-containing derivatives bearing negative charges. Such anionic species might be handled by OATs, which likewise belong to the SLC22A transporter family. Clinical and histological evidence supports this notion, since OATs, like OCTs, are highly expressed in renal tissue, the cochlea, peripheral nerves, and other sites where cisplatin-induced damage is prevalent [15]. Importantly, these same tissues also display expression of copper transporters, raising the unresolved possibility that both SLC22A- and SLC31A-type carriers act in concert to permit platinum drug accumulation to toxic levels [16]. From a therapeutic standpoint, the clinical use of cisplatin is constrained by its narrow effective dose window. Increasing the drug concentration beyond this range in an attempt to overcome tumor resistance is not feasible, as further intracellular accumulation would exacerbate toxicity in healthy tissues. In clinical practice, these limitations are reflected in the cumulative dosing strategies used in oncology. Cisplatin regimens typically aim for cumulative doses of approximately 180–200 mg/m^2^, a range broadly recognized as sufficient to achieve therapeutic benefit while minimizing unacceptable toxicity. Several clinical studies, particularly in head and neck and thoracic malignancies, indicate that cumulative doses of ≥200 mg/m^2^ are associated with improved treatment outcomes, although escalation beyond this level correlates with a markedly increased risk of nephrotoxicity, ototoxicity, and other dose-limiting adverse effects [17,18,19,20,21,22,23,24,25,26]. Incorporating these established dose thresholds underscores the delicate balance between efficacy and toxicity that defines cisplatin’s therapeutic window.

## 3. Molecular Mechanisms of Cisplatin Cytotoxicity

A variety of membrane proteins have been implicated in the trafficking of platinum-based drugs. These include ATP-dependent efflux transporters such as the multidrug resistance-associated proteins (MRPs) and the copper-transporting ATPases (ATP7A and ATP7B), as well as solute carrier systems comprising copper transporters (CTR1), aquaporins (AQP2, AQP9), and additional members of the SLC family. The efflux pump multidrug resistance protein 1 (MDR1), also known as P-glycoprotein and belonging to the ATP-binding cassette superfamily, has long been recognized for its contribution to drug resistance [27,28]. In both yeast and mammalian models, the copper transporter CTR1 has been shown to facilitate cisplatin uptake [29]. Evidence from human cells further demonstrates that exposure to cisplatin accelerates the degradation of CTR1 at the plasma membrane, which diminishes drug influx and thereby promotes resistance [30,31,32]. Functional disruption of CTR1 confers marked resistance to cisplatin in vivo, whereas elevated expression of this transporter increases intracellular platinum content and typically enhances chemosensitivity. Another protein, the transmembrane protein 205 (TMEM205), has been identified as a contributor to platinum resistance. Expression profiling reveals that TMEM205 is enriched in the liver, pancreas, and adrenal glands, and its overexpression in cisplatin-resistant (CP-r) cell lines has been associated with decreased drug sensitivity, suggesting its potential as a therapeutic biomarker or target [27]. In addition, although glucose transporter 1 (GLUT1) does not appear to serve as a direct conduit for cisplatin, aberrant localization of this protein intensifies the resistant phenotype [27,33].

**Figure 2:**
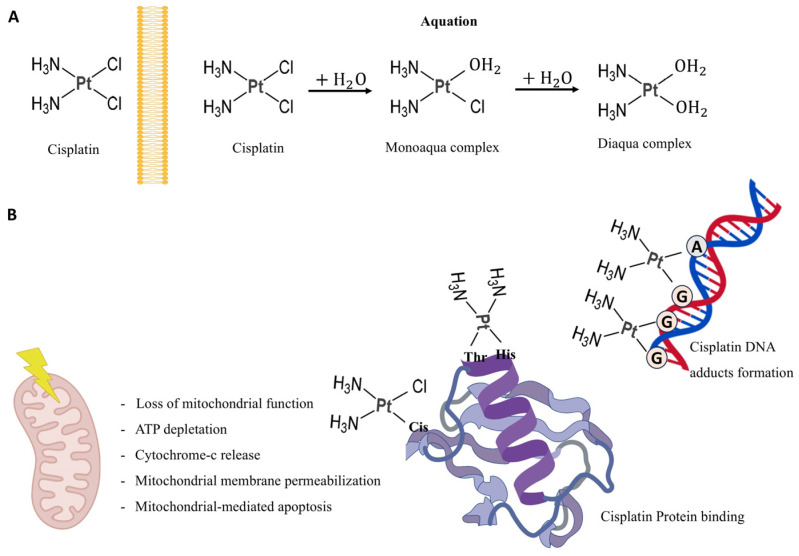
Graphic representation of cisplatin modification and functions. (**A**) Cisplatin acquation process. (**B**) Cisplatin effects on mitochondria and its binding to DNA and proteins. Thr = Thirosine, His = Histidine, Cis = Cisterine, A = Adenine, G = Guanosine. Created with BioGDP.com [34].

Once inside the cytoplasm, cisplatin undergoes aquation in which its chloride ligands are replaced by water molecules, producing highly electrophilic species capable of reacting with cellular nucleophiles (Figure 2).

**Figure 3:**
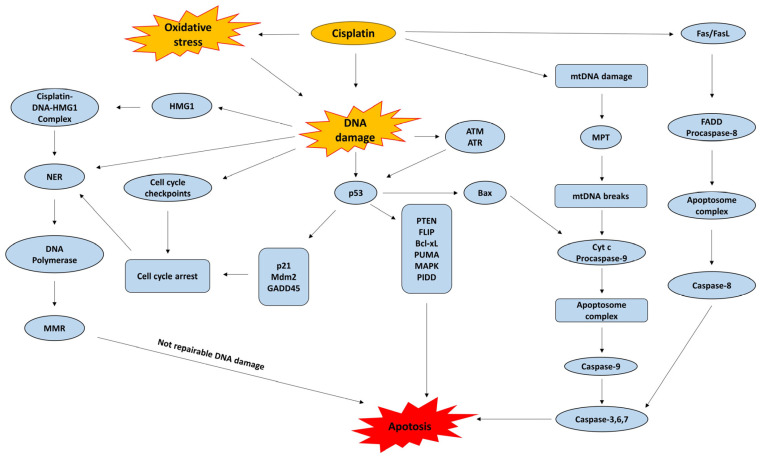
Cisplatin mechanisms of action. NER: Nucleotide Excision Repair; MMR: Mismatch Repair; p53: Tumor protein p53; p21: Cyclin-dependent kinase inhibitor 1 (CDKN1A); Mdm2: Mouse double minute 2 homolog; GADD45: Growth Arrest and DNA Damage-inducible protein 45; HMG1: High-Mobility Group Box 1 protein; ATM: Ataxia Telangiectasia Mutated; ATR: ATM and Rad3-related; PTEN: Phosphatase and Tensin Homolog; FLIP: FLICE-like inhibitory protein; Bcl-xL: B-cell lymphoma—extra large; PUMA: p53 upregulated modulator of apoptosis; MAPK: Mitogen-Activated Protein Kinase; PIDD: p53-induced protein with a death domain; Bax: Bcl-2-associated X protein; mtDNA: Mitochondrial DNA; MPT: Mitochondrial Permeability Transition; Cyt c: Cytochrome c; Fas/FasL: Fas receptor/Fas ligand; FADD: Fas-Associated protein with Death Domain.

These activated forms interact readily with sulfhydryl groups in proteins and with nitrogen atoms in nucleic acids. The primary biological target is DNA, where cisplatin preferentially coordinates with the N7 position of purine bases [35]. The most prevalent lesions are 1,2-intrastrand cross-links involving adjacent guanine residues d(GpG), which account for about 90% of adducts, and adenine-guanine cross-links d(ApG), comprising roughly 10%. Additional DNA distortions include 1,3-intrastrand d(GpXpG) adducts, inter-strand cross-links, and nonproductive binding events, all of which contribute to cytotoxicity. The centrality of DNA as a damage target is underscored by the hypersensitivity to cisplatin observed in organisms lacking proficient DNA repair pathways, spanning both prokaryotic and eukaryotic systems [36,37]. The accumulation of cisplatin-induced DNA lesions initiates a cascade of downstream apoptotic signaling pathways, which constitutes a central mechanistic determinant of its anticancer efficacy (Figure 3).

In healthy cells, intracellular reactive oxygen species (ROS) levels are tightly regulated through a dynamic balance between their generation and clearance. This homeostasis is largely sustained by enzymatic and nonenzymatic defense systems, including reduced glutathione (GSH), superoxide dismutase (SOD), and catalase (CAT) [38]. When this balance collapses under conditions of oxidative stress, excessive ROS accumulation can provoke irreversible injury to macromolecules such as proteins, lipids, and nucleic acids, thereby favoring the onset and progression of several cancerous and non-cancerous diseases [39,40]. Compared with non-malignant cells, tumor cells typically exist in a state of heightened oxidative pressure, a consequence of aberrant signaling cascades, elevated metabolic flux, and defects in mitochondrial function. Among the mechanisms underlying the cytotoxic effects of cisplatin, ROS generation is regarded as a central pathway. Mitochondria are particularly vulnerable to this insult, where cisplatin exposure leads to oxidation of critical sulfhydryl groups in mitochondrial proteins, suppression of calcium uptake, and dissipation of the mitochondrial membrane potential [41].

Oxidative stress disrupts essential cellular processes, and cisplatin contributes to this imbalance by stimulating the production of ROS in addition to its well-established genotoxic activity. The induction of cell death by cisplatin involves the rapid engagement of multiple signaling cascades, the exact nature of which varies depending on the cellular context and tumor type. Both the level of cis-diamminedichloroplatinum(II) exposure and the duration of treatment are critical determinants of the extent of ROS accumulation [42]. The regulation of intracellular redox balance relies heavily on thiol-containing molecules, whose sulfhydryl (-SH) groups play a central role in detoxification. However, under certain conditions, these thiols may undergo oxidation to form thiyl radicals. Such reactive intermediates can subsequently react with molecular oxygen, amplifying the generation of ROS and exacerbating oxidative damage [43].

Elevated concentrations of ROS are capable of initiating programmed cell death through both receptor-mediated (extrinsic) and mitochondria-dependent (intrinsic) mechanisms [44]. Within the extrinsic cascade, ROS production occurs as a proximal signal downstream of Fas ligand engagement, facilitating phosphorylation-dependent activation of Fas. This step is required for recruitment of adaptor molecules such as Fas-associated protein with a death domain and the subsequent activation of caspase-8, ultimately driving apoptosis [45,46]. In the intrinsic pathway, ROS promote the release of cytochrome c from mitochondria by interfering with the regulatory balance of Bcl-2 family proteins—enhancing the activity of pro-apoptotic members such as Bax and Bak while compromising the function of anti-apoptotic counterparts including Bcl-2 and Bcl-xL [47,48,49]. At even higher intracellular ROS burdens, cells may undergo not only apoptosis but also necrosis, reflecting overwhelming oxidative injury [50]. Furthermore, ROS have been implicated in triggering autophagy, a catabolic survival-to-death mechanism characterized by the sequestration of damaged organelles and protein aggregates into autophagosomes for lysosomal degradation [51].

In addition to apoptosis, increasing evidence indicates that cisplatin can trigger multiple forms of regulated necrosis, including ferroptosis, necroptosis and pyroptosis, depending on the cellular context and the intensity of oxidative and metabolic stress [52].

Pyroptosis is an inflammatory form of programmed cell death orchestrated by gasdermin (GSDM) family proteins, particularly GSDMD and GSDME, whose activation culminates in membrane pore formation and the release of proinflammatory cytokines [53]. Central to this mechanism is the NLRP3 inflammasome, a complex of NLRP3, ASC, and pro-caspase-1, which governs caspase-mediated cleavage of gasdermins and thereby initiates pore formation and cell lysis [54]. Cisplatin has emerged as a potent upstream driver of this pathway in the inner ear, where it stimulates NLRP3 activation, enhances GSDMD/GSDME processing, and accelerates pyroptotic death in cochlear sensory and supporting cells [55,56]. Thus, reduction in pyroptosis may induce cisplatin resistance in neoplastic cells [57,58,59].

Ferroptosis, an iron-dependent, lipid peroxidation-driven form of regulated necrosis, is increasingly recognized as a contributor to cisplatin toxicity. By depleting glutathione and impairing mitochondrial redox balance, cisplatin promotes accumulation of lipid hydroperoxides, thereby lowering the threshold for ferroptotic death [60]. A recent study demonstrated that triggering ferroptosis can counteract cisplatin resistance in gastric cancer. Increased ATF3 expression drives ferroptotic cell death by inhibiting the Nrf2–Keap1–xCT pathway, thereby limiting tumor progression and enhancing cisplatin sensitivity in gastric cancer cells [61].

Necroptosis is a regulated cell death program that shares morphological features with unregulated necrosis but is driven by a defined molecular apparatus distinct from apoptosis. Its core effector module consists of RIP1, RIP3, and MLKL, which assemble into a cytotoxic complex that disrupts membrane integrity and promotes inflammatory mediator release [62]. Cisplatin acts as a potent upstream initiator of this cascade, particularly within renal proximal tubular cells where the drug preferentially accumulates [63]. Increasing intracellular cisplatin burden intensifies necroptotic signaling, elevates RIP1 and RIP3 activity, and amplifies cytokine production, thereby accelerating tissue injury and contributing to cisplatin-induced acute kidney injury [64]. Targeted suppression of necroptosis, either via deletion of RIP1, RIP3, or MLKL or through pharmacologic inhibition, markedly attenuates cisplatin-associated renal toxicity without diminishing its anticancer efficacy [65]. This direct functional linkage positions the necroptotic pathway as a central mediator of cisplatin nephrotoxicity. Cisplatin-driven necroptosis also intersects with apoptosis and autophagy, revealing a broader regulatory network. The proapoptotic signaling of cisplatin can impair autophagic flux, reducing a key survival mechanism and increasing reliance on necroptotic cell death.

Collectively, these alternative programmed necrotic pathways do not replace apoptotic signaling but operate in parallel or downstream of it, particularly under conditions of severe oxidative stress [60,66,67]. Their contribution may enhance antitumor cytotoxicity but also exacerbate organ-specific toxicity, reinforcing the complexity of the cell-death landscape of cisplatin.

When intracellular chloride concentration is low, cisplatin undergoes aquation, producing highly reactive ionic species such as the monoaqua complex [cis-(NH_3_)_2_PtCl(H_2_O)]^+^ and the diaqua complex [cis-(NH_3_)_2_Pt(H_2_O)_2_]^2+^ [68,69,70]. These hydrolyzed derivatives exhibit reactivities up to a thousand-fold greater than the parent drug, largely due to their ability to interfere with mitochondrial oxidative metabolism by uncoupling oxidative phosphorylation [71]. This mitochondrial dysfunction is accompanied by calcium efflux, leading to transient elevations in cytosolic calcium concentration, which severely perturbs calcium homeostasis and compromises normal cellular physiology.

Mitochondrial glutathione (GSH) plays a central role in sustaining redox balance and preserving enzymatic thiol groups in their reduced, active state. Loss of this protective system results in irreversible enzyme inactivation. Cisplatin toxicity, especially in renal tissue, has been linked to depletion of both GSH and protein-associated sulfhydryl groups. Nicotinamide adenine dinucleotide (NADH), another crucial cofactor required for maintaining reduced thiols, also decreases after cisplatin exposure. The combined reduction of GSH and NADH compromises mitochondrial dehydrogenase activity, uncouples oxidative phosphorylation, and promotes hydroxyl radical formation, thereby amplifying oxidative stress [72]. These reactive species initiate the peroxidation of polyunsaturated lipids and structural proteins, leading to a self-amplifying lipid peroxidation cascade that ultimately results in irreversible damage to mitochondrial and cellular membranes [73,74].

Ultimately, disruption of calcium balance and oxidative injury converge to drive key pathological events, including loss of mitochondrial function, ATP depletion, impairment of vital cofactors, and collapse of energy metabolism. These processes culminate in apoptotic signaling and, in severe cases, necrotic cell death. Interestingly, modulation of intracellular calcium levels through exogenous supplementation has been proposed as a possible protective strategy, since calcium may compete with cisplatin for binding sites and thereby attenuate certain toxic side effects [35,75].

Programmed cell death via apoptosis is a highly regulated molecular process that requires energy and is marked by specific structural transformations, including nuclear condensation, cell shrinkage, external display of phosphatidylserine, and membrane blebbing. The central regulators of this cascade are caspases, a conserved protease family that cleaves substrates after aspartate residues [76,77]. Caspases are subdivided into initiators, which include caspase-8 and caspase-9, and executioners, such as caspase-3 and caspase-7. Initiator caspases function as sensors that respond to upstream apoptotic stimuli: caspase-8 is recruited and activated at plasma membrane death receptors, while caspase-9 is activated within the apoptosome after mitochondrial cytochrome-c release. Once activated, executioner caspases dismantle the cell by targeting key nuclear and cytoplasmic proteins, such as PARP and inhibitors of caspase-activated DNases [78,79].

Cisplatin is particularly effective in driving apoptosis, and failure of apoptotic signaling has been directly associated with the emergence of drug resistance. Apoptosis can proceed through two primary molecular routes [80]. The extrinsic pathway begins when ligands of the TNF-α receptor superfamily bind to their cognate receptors, leading to receptor clustering and recruitment of adaptor proteins that nucleate the death-inducing signaling complex (DISC). This complex facilitates procaspase-8 activation, thereby launching the apoptotic cascade. The intrinsic pathway, in contrast, is triggered by intracellular stresses such as cisplatin-induced DNA lesions, which compromise mitochondrial integrity and provoke cytochrome-c efflux. Cytochrome-c then interacts with APAF-1 to assemble the apoptosome, resulting in procaspase-9 activation. The balance of pro-apoptotic (e.g., Bax, Bak) and anti-apoptotic (e.g., Bcl-2, Bcl-xL) Bcl-2 family proteins dictates the release of cytochrome-c, thus serving as a decisive checkpoint for mitochondrial apoptosis [81]. Cisplatin activates a spectrum of stress-responsive signaling networks, and the cellular outcome—either apoptotic death or the acquisition of resistance—depends on how these pathways are integrated and executed [80,82].

Protein kinase C (PKC) represents a group of phospholipid-activated enzymes that phosphorylate numerous downstream substrates and thereby influence cell growth, apoptosis, and stress signaling [83,84]. The functional contribution of PKC to cisplatin response remains controversial: in some cellular models, PKC activation enhances susceptibility to cisplatin, while in others, drug sensitivity requires PKC inhibition or downregulation [85,86,87,88]. These divergent outcomes likely reflect differences in isozyme-specific signaling. Distinct PKC isoforms can modulate pathways related to apoptosis, DNA repair capacity, and drug transport, meaning that the effect of PKC activity on cisplatin resistance is not universal but instead depends on which isoforms are dominant in a given cellular context [83,89].

Mitogen-activated protein kinases (MAPKs) constitute a conserved family of serine/threonine kinases that integrate extracellular stimuli to control processes such as proliferation, differentiation, and survival [90,91,92]. Cisplatin exposure has been consistently linked to activation of the extracellular signal-regulated kinase (ERK) pathway, though experimental outcomes differ on whether ERK activity promotes resistance to drug-induced apoptosis or instead enhances cisplatin-mediated cytotoxicity [93,94,95,96,97].

Notably, ERK activation occurs upstream of p53 signaling: ERK directly phosphorylates p53, resulting in transcriptional induction of target genes such as p21, growth arrest and DNA damage-inducible 45 (GADD45), and Mdm2 [98]. Through these interactions, ERK activation can enforce cell cycle arrest, which provides an interval for repair of cisplatin-induced DNA lesions in a p53-dependent manner.

Cellular stress responses strongly influence cisplatin-triggered apoptosis, in large part through activation of the p38 MAPK signaling cascade. One identified downstream target of this pathway is the epidermal growth factor receptor (EGFR), whose internalization upon cisplatin exposure occurs following p38-dependent phosphorylation [99]. p38 MAPK also exerts pro-apoptotic effects through regulation of the adaptor protein p18, which forms complexes with p53 and promotes transcription of the pro-death genes PUMA and NOXA [100]. Collectively, these findings underscore the pivotal role of p38 MAPK as a mediator of cisplatin-induced apoptotic signaling.

The c-Jun N-terminal kinase (JNK), also referred to as stress-activated protein kinase, is a central effector of signaling pathways triggered by environmental and genotoxic stressors. DNA damage, including lesions produced by platinum-based agents, serves as a potent activator of JNK. Both stereoisomeric forms of cisplatin (cis and trans) have been shown to stimulate JNK signaling cascades, highlighting the pathway’s broad responsiveness to platinum-induced stress [101]. A pivotal link between JNK activity and apoptosis is mediated through p73, a pro-apoptotic transcription factor and structural homolog of p53. Upon activation, p73 physically associates with JNK, forming a functional complex that enhances transcription of death-promoting genes, thereby reinforcing cisplatin-induced cell death [102,103]. Beyond its interaction with p73, JNK also interfaces with mitochondrial apoptotic regulators, modulating the activity of Bcl-2 family proteins and facilitating cytochrome c release. In parallel, JNK contributes to death receptor signaling, where it amplifies caspase activation and apoptosis induction. These converging inputs position JNK as an essential signaling hub that integrates nuclear DNA damage with both extrinsic and intrinsic apoptotic machinery during cisplatin treatment.

Akt, a serine/threonine kinase downstream of phosphoinositide 3-kinase (PI3K), is a central regulator of prosurvival signaling [104]. Following cisplatin exposure, DNA damage has been shown to activate Akt-dependent phosphorylation of the pro-apoptotic Bcl-2 family protein BAD at serine-136 [105]. This modification of BAD is observed in both cisplatin-sensitive and -resistant tumor cells, and its phosphorylation is essential to sustain viability after drug treatment. Mechanistically, BAD regulation involves dual input from distinct kinase pathways: ERK signaling mediates phosphorylation at serine-112, while PI3K/Akt controls phosphorylation at serine-136. Inhibition of either cascade sensitizes ovarian carcinoma cells to cisplatin, underscoring the cooperative role of ERK and Akt in converging on BAD to neutralize its apoptotic function [105].

Cisplatin response also engages the p53 axis. p53, a transcription factor with a normally short half-life, is stabilized and activated through phosphorylation by the ATM kinase in response to DNA strand breaks. Once activated, p53 induces Mdm2, an E3 ubiquitin ligase that contributes to feedback regulation of its stability. Beyond this autoregulatory loop, p53 transactivates numerous genes involved in DNA repair, cell cycle arrest, and apoptosis. In cisplatin-treated cells, p53-dependent death pathways include degradation of the anti-apoptotic FLIP protein, direct antagonism of Bcl-xL function, induction of PTEN, and suppression of AMPK signaling [106]. Interestingly, cisplatin cytotoxicity is not limited to p53-proficient cells; p53-deficient systems can still undergo apoptosis upon treatment. This indicates that alternative, p53-independent mechanisms exist to mediate stress responses and cell death following cisplatin-induced DNA lesions.

## 4. Mechanisms of Cisplatin Resistance

**Figure 4:**
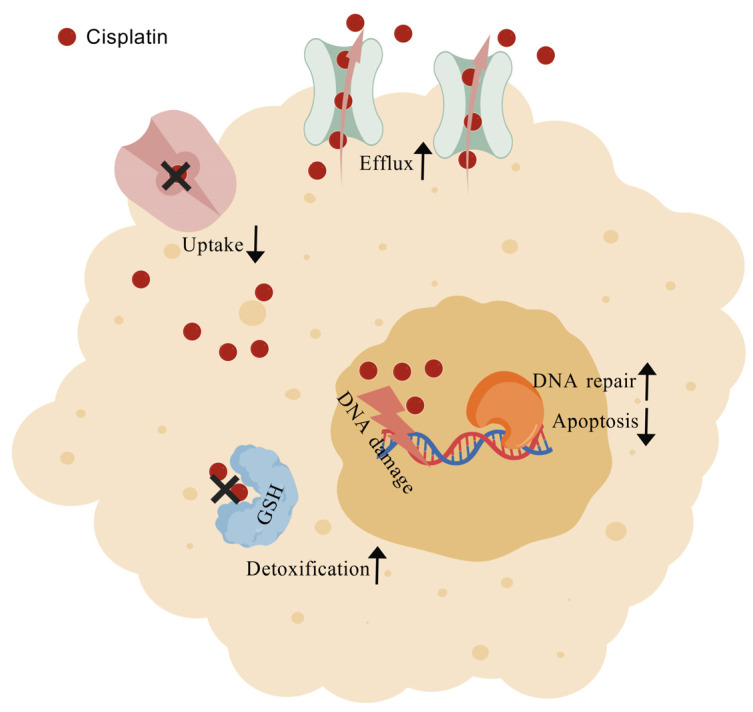
Graphic representation of cisplatin resistant mechanisms. Created with BioGDP.com [34].

One of the biggest challenges in oncology remains the emergence of resistance to platinum-based chemotherapeutics such as cisplatin. Alongside dose-limiting toxicities, this phenomenon continues to undermine the efficacy of these drugs in the management of solid tumors. Experimental data from in vitro models indicate that resistance to cisplatin can arise through diverse epigenetic and cellular modifications. Mechanisms include diminished intracellular drug accumulation—achieved through active efflux, intracellular sequestration, or impaired uptake—together with detoxification by glutathione conjugates, metallothioneins, and other antioxidant systems (Figure 4). Additional resistance routes involve elevated DNA repair capacity via nucleotide excision and mismatch repair pathways, aberrant DNA methylation, altered membrane trafficking due to cytoskeletal disorganization, overexpression of molecular chaperones, dysregulated expression of microRNAs, and changes in transcription factors or small GTPases [107]. A consequence of reduced cisplatin uptake or retention is a substantial decrease in platinum–DNA adduct formation, which undermines cytotoxic efficiency. For instance, cisplatin-resistant hepatoma 7404-CP20 cells display up to a nine-fold reduction in overall platinum–DNA adducts and a twelve-fold reduction in rRNA gene-specific cross-links, despite removal kinetics comparable to parental 7404 cells [108]. Cisplatin also provokes endoplasmic reticulum stress and apoptotic signals independent of nuclear involvement [109]. Since the drug functions as both a DNA-damaging agent and apoptosis inducer, alterations in heat-shock protein (HSP) expression are strongly implicated in resistance. Elevated HSP60 has been detected in cervical and liver carcinoma [27], while HSP27, HSP70, HSP72, GRP78, and HSP90 upregulation has been documented in resistant ovarian [110,111], cervical [112], colon [113], breast [114], and laryngeal carcinoma models [42].

Members of the Rho family of small GTP-binding proteins—RhoA, RhoB, RhoC, and Rac—also contribute to cisplatin resistance. Reduced expression of Rab5, Rac1, and RhoA correlates with impaired accumulation of radiolabeled cisplatin in resistant cells [107]. Specific ribosomal proteins are likewise implicated: L36 has been linked with resistance in carcinoma cells [115], while proteomic analysis in MCF-7 breast cancer cells revealed decreased P0 expression [116], and ectopic expression of L37 can blunt p53-mediated DNA damage responses [117]. Transcriptional regulators also play prominent roles, including Y-box binding protein-1, CCAAT-binding factor 2, activating transcription factor 4, zinc finger protein 143, nuclear factor kappa-light-chain-enhancer of activated B cells (NF-κB), microphthalmia-associated transcription factor (MITF), forkhead box O (FoxO), and the mitochondrial transcription factor A (TFAM) [27]. Nuclear factor erythroid 2-related factor 2 (Nrf2) is a key transcription factor that plays a key role antioxidant response and is involved in the onset and progression of several cancerous and non-cancerous diseases [118,119,120] and is emerging as a pivotal determinant: Nrf2-deficient murine embryonic fibroblasts and cisplatin-resistant SK-OV ovarian cancer cells display a clear correlation between Nrf2 levels and drug resistance. Silencing or loss of Nrf2 enhances cisplatin cytotoxicity and apoptosis compared with controls [121]. This effect of Nrf2 in inducing cisplatin resistance and cancer progression has been demonstrated in several malignancies [122,123,124]. Finally, upregulation of GCF2 in KB-3-1 cells reduces RhoA expression, perturbs actin–filamin organization, causes mislocalization of the efflux transporter MRP1, and lowers cisplatin accumulation, collectively producing a three-fold increase in drug resistance [27].

Evidence suggests that alterations in several signaling cascades contribute to the acquisition of cisplatin resistance. These include modifications in protein kinase C isoforms and activation of MAPK or small GTP-binding proteins and such changes may arise directly from drug exposure or through drug-independent adaptive responses. More recently, a mechanism involving the nuclear translocation of the glucocorticoid receptor following cisplatin binding has been described. This event upregulates MAST1, a kinase that promotes platinum resistance by reactivating MEK1 via phosphorylation at Ser221 [125]. The result is inhibition of apoptotic signaling and enhanced cellular proliferation. The clinical relevance of this pathway is underscored by the observation that pharmacological inhibition of MAST1 with agents such as lestaurtinib reinstates apoptosis and decreases tumor growth [126].

At the molecular level, epigenetic reprogramming contributes substantially to resistance phenotypes. Mechanisms include increased detoxification through glutathione, metallothioneins, or antioxidant conjugates, altered trafficking of membrane proteins, rearrangements of cytoskeletal structures, and deregulation of transcription factors and small GTPases (Figure 4). Platinum sequestration, reduced intracellular uptake, and enhanced efflux also belong to this spectrum. Although robust preclinical and clinical data support reduced uptake and enhanced efflux as contributors to resistance, the clinical significance of detoxification-mediated inactivation remains less definitive [27]. To classify these mechanisms systematically, Galluzzi and colleagues proposed their subdivision into pre-target, on-target, post-target, and off-target categories [127]. Pre-target events encompass reduced drug import, increased export, and enhanced conjugation to detoxifying molecules such as glutathione and metallothioneins. On-target mechanisms primarily involve alterations in DNA damage processing. These include elevated nucleotide excision repair activity, defects in mismatch repair, upregulation of homologous recombination, and increased tolerance to DNA adducts through translesion synthesis (Figure 4). In fact, the polymerases POLH, POLI, POLK, REV1, REV3, and REV7 cooperate in translesion synthesis to bypass DNA crosslinks. Experimental data reveal that loss of POLH or REV3 sensitizes tumor cells to cisplatin, whereas REV3 overexpression has the opposite effect, underscoring the therapeutic potential of modulating this pathway [128,129]. In addition, the possibility that cytoplasmic proteins may sequester cisplatin and thereby attenuate its DNA-damaging effects remains underexplored. Post-target resistance is associated with signaling alterations downstream of DNA damage that regulate apoptosis. Normally, cisplatin-induced inter- and intra-strand crosslinks activate apoptotic cascades, but mutations or epigenetic changes in these pathways confer survival advantages. MAPK and p53 signaling are among the most frequently implicated routes. Notably, p53 dysfunction explains the paradoxical observation that tetraploid cells often exhibit greater tolerance to DNA-damaging agents compared with diploid counterparts [130]. Dysregulation of apoptosis-related proteins, such as the upregulation of survivin via a PI3K/AKT1-dependent pathway, has been correlated with diminished cisplatin sensitivity and poor patient survival in multiple malignancies, including ovarian and esophageal cancers [131]. Off-target mechanisms include signaling networks not directly engaged by cisplatin yet capable of compensating for its cytotoxic effects. Key examples are the dual-specificity kinase DYRK1B, which participates in differentiation programs, and ERBB2/HER2, which activates pro-survival pathways through MAPK and PI3K/AKT. Glutathione again emerges as a central player, as it modulates not only pre-target detoxification but also redox balance, thereby suppressing apoptosis and enhancing tolerance to oxidative stress. This places glutathione at the intersection of multiple resistance pathways [127].

Finally, increasing attention has been directed toward the role of STAT3 signaling, a key pathway involved in the onset and progression of several diseases [132,133]. In fact, it has been demonstrated that STAT3 contributes to tumor cell survival, proliferation, angiogenesis, immune evasion, and importantly, drug resistance. Persistent activation of STAT3, for instance via S1PR1 upregulation as observed in gastric cancer, leads to constitutive cisplatin resistance [134]. Inhibition of the S1PR1–STAT3 signaling loop has been shown to restore drug sensitivity, highlighting this axis as a potential therapeutic target to overcome resistance [135].

## 5. Therapeutic Applications of Cisplatin in Oncology

### 5.1. Ovarian Cancer

Among gynecologic malignancies, ovarian carcinoma represents one of the most prevalent and lethal forms of disease in women, with mortality rates surpassing those of other cancers affecting the female reproductive tract [136,137]. Although the precise molecular and environmental determinants of ovarian tumorigenesis remain unresolved, familial predisposition—particularly in patients with a history of breast or colorectal cancer—appears to play an important contributory role [123,138]. A major clinical challenge is the absence of reliable screening programs and the nonspecific nature of early manifestations, which frequently leads to late-stage diagnosis. Standard management typically involves surgical resection of the tumor mass, followed by systemic chemotherapy to eradicate residual microscopic disease [139]. Despite dose-limiting toxicities, cisplatin continues to be regarded as a cornerstone chemotherapeutic agent in ovarian cancer therapy. However, one of its major limitations lies in the propensity of tumors to recur and to acquire resistance following initial therapeutic response. To counteract this phenomenon, cisplatin is increasingly administered in combinatorial regimens with bioactive agents such as honey venom, trichostatin A or 5-aza-2′-deoxycytidine, and withaferin A, which aim to enhance antitumor efficacy while reducing the likelihood of resistance [140,141,142]. In addition, many other synthetic and natural molecules are being investigated for their ability to synergistically enhance cisplatin cytotoxicity [143,144,145,146].

### 5.2. Breast Cancer

Breast carcinoma remains a principal contributor to cancer-related mortality among women on a global scale. In advanced or highly aggressive forms of the disease, chemotherapy constitutes the primary therapeutic intervention, with the objective of prolonging patient survival [147]. Over the years, multiple chemotherapeutic compounds have been introduced in an attempt to overcome the persistent challenge posed by breast cancer [148]. The majority of these pharmacological agents act by indiscriminately targeting populations of rapidly proliferating cells, a mechanism that results in significant collateral tissue damage and accounts for their classification as cytotoxic drugs. Within this group, cisplatin has achieved prominence as a versatile antineoplastic agent, demonstrating clinical utility. Cisplatin exerts its antitumor activity in breast cancer primarily through the formation of covalent DNA adducts that disrupt replication and transcription, producing replication fork stalling and extensive genomic stress that exceed the capacity of tumor cells to repair the damage. This DNA injury serves as the central initiating event for activation of the intrinsic apoptotic pathway, where p53 signaling, induction of BH3-only proteins, and mitochondrial outer-membrane permeabilization converge to drive caspase activation and apoptotic cell death. Tumors with defects in high-fidelity DNA repair, particularly homologous recombination, display heightened susceptibility to cisplatin-induced cytotoxicity; the article highlights, for example, the critical role of BRCA1/2 and RAD51-mediated repair in determining whether cells can tolerate platinum-induced DNA crosslinks or instead undergo apoptosis [149]. Beyond direct genotoxicity, cisplatin affects multiple stress-response pathways that modulate treatment outcome. Activation of the MAPK family members JNK and p38 enhances pro-apoptotic signaling under sustained stress, whereas ERK activation can promote survival, underlying how pathway-specific signaling biases influence cellular fate following platinum exposure. Finally, metabolic state also impact cisplatin efficacy since glycolytic reprogramming and elevated antioxidant capacity can buffer the oxidative stress generated downstream of cisplatin, whereas impaired redox homeostasis sensitizes cancer cells to platinum-induced damage [149,150].

### 5.3. Lung Cancer

Lung cancer continues to rank among the most prevalent and deadly forms of malignancy worldwide. Small-cell lung carcinoma (SCLC) accounts for approximately 15% of all diagnosed cases, and the current therapeutic backbone for this subtype relies heavily on platinum-based regimens [151]. Among these, cisplatin and carboplatin remain the principal agents utilized in chemotherapy protocols for SCLC [152]. Cisplatin is frequently favored in clinical trials owing to its potent antineoplastic properties; however, its clinical application is often limited by severe toxicities, notably nephrotoxicity [153,154] and gastrointestinal side effects such as nausea and emesis [155]. To mitigate renal complications, cisplatin administration requires rigorous hydration strategies and careful monitoring of urine output. Given these challenges, carboplatin is frequently adopted as an alternative, offering comparable efficacy while circumventing the need for aggressive fluid management.

For patients with localized non-small-cell lung cancer (NSCLC), surgical resection constitutes the standard therapeutic approach, with adjuvant cisplatin-based chemotherapy recommended in stage II and III disease. The Lung Adjuvant Cisplatin Evaluation (LACE) meta-analysis, which pooled data from the five largest randomized trials, demonstrated a 5.3% absolute improvement in 5-year overall survival following cisplatin-based adjuvant therapy, reinforcing the value of this approach [156].

In addition to treatment strategies, tumor biology has highlighted cluster of differentiation 133 (CD133), a membrane glycoprotein associated with stem cell phenotypes, as a putative marker of tumor-initiating cells. Notably, studies have reported that populations expressing both CD133 and epithelial-specific antigen (ESA) are significantly enriched in primary NSCLC tissues compared to normal lung counterparts [157].

### 5.4. Head and Neck Squamous Cell Carcinoma

Head and neck squamous cell carcinoma (HNSCC) remains a prevalent and aggressive form of cancer, with global incidence figures exceeding 600,000 new diagnoses annually and may be preceded by potentially malignant disorders [158,159]. Despite advancements in therapeutic approaches such as surgical intervention, radiotherapy, and chemotherapeutic regimens, the disease continues to carry a poor prognosis [160,161]. The overall survival rate at five years has remained stagnant at around 50%, underscoring the persistent clinical challenges in its management [162].

While cisplatin is commonly incorporated into treatment strategies, its effectiveness as a monotherapy for HNSCC has been limited [162,163,164]. To enhance therapeutic outcomes, researchers have explored combination therapies involving cisplatin and other agents, such as methotrexate, vinblastine, doxorubicin, and gemcitabine. Comparative clinical studies investigating these combinations—particularly in metastatic urothelial cancer contexts—have been pivotal in evaluating the efficacy of multi-drug protocols over single-agent cisplatin use. In HNSCC, the acquisition of stem-like characteristics has been strongly correlated with the upregulation of markers such as SOX2, Bmi-1, CD44, Nanog, and aldehyde dehydrogenase. The presence of these markers defines a subpopulation of cells that demonstrates heightened tolerance to cisplatin therapy [165]. This phenomenon highlights a critical feature of cancer stem cells (CSCs) in the context of therapeutic resistance, and as a result, extensive research efforts have been directed toward elucidating the functional significance of these stemness-associated markers in cisplatin-resistant HNSCC [166,167,168].

### 5.5. Glioblastoma Multiforme

Glioblastoma multiforme (GBM) is the most aggressive and frequently diagnosed primary malignant tumor of the central nervous system, characterized by diffuse infiltration, rapid progression, and almost inevitable recurrence [169]. Despite advances in surgical techniques and radiotherapy, prognosis remains dismal, with median overall survival rarely exceeding 15–18 months even under the current standard of care combining surgery, radiotherapy, and temozolomide [170]. Given its broad antitumor efficacy across solid malignancies, cisplatin has been explored as an adjunct or salvage chemotherapeutic agent in GBM, particularly in recurrent disease or pediatric brain tumors. In the context of GBM, cisplatin has been administered both as a single agent and in combination regimens, often with alkylating agents, topoisomerase inhibitors, or novel radiosensitizers, in an attempt to overcome the intrinsic chemoresistance of glioma cells [171]. Nevertheless, systemic dosing is frequently limited by toxicity, necessitating careful hydration protocols and renal function monitoring to minimize nephrotoxic risk.

One of the greatest challenges in applying cisplatin for GBM lies in the intrinsic and acquired resistance mechanisms of the tumor. Glioblastoma cells exhibit upregulated DNA repair pathways, particularly nucleotide excision repair and mismatch repair components, which facilitate the removal of cisplatin-induced DNA adducts. Overexpression of the DNA repair enzyme O6-methylguanine-DNA methyltransferase (MGMT), well known for its role in resistance to temozolomide, has also been implicated in reducing cisplatin cytotoxicity [172]. Additionally, glioma stem-like cells (GSCs) display enhanced drug efflux through ATP-binding cassette (ABC) transporters, elevated antioxidant defenses, and activation of survival signaling cascades such as PI3K/AKT and NF-κB, further diminishing cisplatin sensitivity. These resistance mechanisms collectively explain why clinical responses to cisplatin in GBM are often transient and incomplete [173].

Toxicity remains another major limitation of cisplatin therapy in glioblastoma [174]. In neuro-oncology, adverse events are particularly problematic because patients often require prolonged treatment courses and are already debilitated by neurological deficits. As a result, carboplatin, a less nephrotoxic analog, has occasionally been employed as a substitute, though evidence suggests it may offer reduced potency in GBM.

### 5.6. Skin Cancers

Cisplatin has been investigated in the treatment of both melanoma and non-melanoma skin cancers (NMSC). In melanoma, cisplatin was among the earliest chemotherapeutic agents to show in vitro cytotoxicity; however, clinical outcomes have been disappointing, as the disease is largely refractory to platinum-based therapy [175,176]. Multiple mechanisms underlie this resistance, including enhanced DNA repair through nucleotide excision repair (NER), upregulation of survival signaling pathways such as PI3K/AKT and MAPK, increased expression of anti-apoptotic proteins, and drug efflux mediated by ATP-binding cassette (ABC) transporters. Clinical regimens incorporating cisplatin have demonstrated transient responses but without significant improvements in overall survival. By contrast, in non-melanoma skin cancers, particularly cutaneous squamous cell carcinoma (cSCC), cisplatin has retained a more prominent role. Cisplatin is frequently administered in combination with 5-fluorouracil (5-FU), and is also utilized as a radiosensitizer in concurrent chemoradiation protocols, especially for advanced head and neck squamous cell carcinoma, which shares biological features with cSCC. Response rates in this context are significantly higher than those observed in melanoma, although resistance and toxicity still limit long-term efficacy [177].

### 5.7. Gastric Cancer

Gastric cancer, arising from the epithelial lining of the gastric mucosa, represents a major malignancy of the digestive system and it ranks as the fifth most prevalent cancer globally, with an incidence of 5.6%, and is responsible for 7.7% of cancer-related mortality [178]. Cisplatin exerts its cytotoxic activity in gastric cancer primarily through the generation of intra- and interstrand DNA crosslinks that impede replication and transcription, imposing a genotoxic burden that often surpasses the repair capacity of malignant cells [179]. This damage activates the DNA damage response, leading to replication fork collapse, checkpoint activation, and engagement of intrinsic apoptotic pathways when repair mechanisms are overwhelmed. Mitochondrial dysfunction and oxidative stress further amplify this response, reinforcing the apoptotic cascade [179]. Effective cisplatin activity therefore depends on the integrity of pro-apoptotic machinery, including mitochondrial permeabilization, cytochrome c release, and caspase activation. Gastric tumors exhibiting intact apoptotic signaling are more susceptible to cisplatin, whereas those with upregulated anti-apoptotic factors or survival pathways such as ERK, NF-κB, or PI3K/Akt, can diminish apoptotic output and reduce drug sensitivity [180,181,182,183,184,185].

In advanced gastric cancer, the integration of chemotherapy with targeted therapeutic approaches has been shown to extend overall survival and enhance patient quality of life. Among the chemotherapeutic agents employed, cisplatin has long served as a cornerstone in first-line regimens [186]. Nonetheless, the therapeutic benefits of cisplatin are frequently undermined by the emergence of drug resistance and the substantial toxicity profile, both of which remain critical barriers to improving long-term outcomes in gastric cancer management [187,188].

### 5.8. Testicular Cancer

Cisplatin itself was formally approved by the FDA in 1978 for use against testicular malignancies [189]. Testicular cancers are classified into seminomas and non-seminomas, both of which primarily affect young men and, despite their shared germ cell origin, these entities differ markedly in differentiation status, biological behavior, and therapeutic responsiveness. Seminomas can arise across a wide range of ages, but they usually progress and metastasize more gradually compared to non-seminomas. At the molecular level, seminomas are characterized by limited DNA damage repair capacity and a pronounced propensity to undergo apoptosis following genotoxic stress. Standard management of seminomas relies on cisplatin-based chemotherapy, with approximately 85% of patients with advanced disease achieving remission after three to four treatment cycles [190]. The exceptional sensitivity of seminomas to cisplatin has been attributed to inefficient nucleotide excision repair and an intact p53-mediated apoptotic response, which together amplify the cytotoxic impact of platinum-induced DNA crosslinks [191].

Non-seminomas are more frequently diagnosed in men from their late teens to early thirties and consist of four main histological variants: embryonal carcinoma, yolk sac tumor, choriocarcinoma, and teratoma. These components often coexist within the same tumor, contributing to biological heterogeneity and variable treatment responses. Among these, teratomas are most effectively treated with combination therapy incorporating bleomycin, etoposide, and cisplatin, producing cure rates above 90% [192]. While most non-seminomatous elements are highly chemosensitive, teratomatous components display intrinsic resistance to cisplatin and frequently require surgical resection to achieve durable disease control [191]. Overall, the extraordinary clinical success of cisplatin in testicular cancer reflects a convergence of biological vulnerabilities, including impaired DNA repair, low tolerance for replication stress, and robust activation of apoptotic pathways, which collectively distinguish germ cell tumors from most other solid malignancies.

### 5.9. Bladder Cancer

Bladder cancer represents the tenth most prevalent malignancy worldwide, with an estimated global incidence of 573,000 new diagnoses and approximately 213,000 disease-related deaths annually [178,193]. Since their introduction in the late 1980s, cisplatin-based combination regimens have constituted the standard adjuvant therapeutic approach for patients with metastatic bladder cancer, and cisplatin continues to serve as a fundamental component of systemic treatment [194]. Despite this, clinical efficacy remains restricted, with objective responses observed in only about 35% of metastatic cases. Furthermore, even among initially responsive patients, the eventual acquisition of cisplatin resistance is almost inevitable [194,195]. This phenomenon of drug resistance is recognized as a principal determinant underlying the poor overall prognosis associated with advanced bladder cancer.

## 6. Cisplatin-Induced Organ Toxicity

A major limitation of cisplatin therapy is the broad spectrum of toxicities it induces. These effects arise directly from the covalent interaction of cisplatin (and carboplatin) with DNA purine residues, as well as indirectly from drug-triggered increases in oxidative stress once endogenous scavenging defenses become engaged in adduct formation. Although virtually any organ can be susceptible to injury given the generalized mode of action, the liver, kidney, heart, auditory system, and peripheral nerves are among the most consistently affected. Despite considerable progress in delineating the cellular and molecular mechanisms by which cisplatin, carboplatin, and oxaliplatin exert their toxic effects, predicting the specific organs at risk in a given patient remains challenging. The severity and pattern of organ involvement are highly variable, and few clinical trials have addressed these questions due to inherent difficulties such as heterogeneous patient populations, differences in treatment regimens, and limitations in control groups or blinding strategies. These same challenges extend to evaluating adjunctive therapies intended to mitigate toxicity. Magnesium supplementation has shown some promise in reducing the incidence of acute kidney injury and oliguria, but more comprehensive evidence is needed to establish its protective efficacy [196].

Hepatic injury is frequently associated with high-dose cisplatin [197]. Mechanistically, hepatotoxicity is linked to oxidative stress characterized by depletion of glutathione and the production of reactive aldehydes, including malondialdehyde, generated via CYP2E1-mediated lipid peroxidation [198]. In such cases, serum enzymes—transaminases and alkaline phosphatase—remain the most sensitive markers of parenchymal and biliary damage, whereas conjugated and unconjugated bilirubin levels provide functional assessment of hepatic impairment [199].

Cardiotoxicity is another clinically relevant concern. Reliable indicators of myocardial damage include elevated cytosolic lactate dehydrogenase and creatine kinase, with increases in serum troponin C signaling irreversible cardiomyocyte necrosis [200].

The kidney represents the primary route of cisplatin elimination and, consequently, a major target of injury. Renal accumulation of cisplatin can reach concentrations several times higher than plasma levels, particularly within proximal tubular epithelial cells where uptake is mediated by copper transporter CTR1 and organic cation transporter OCT2 [29,201]. The clinical manifestation is acute kidney injury, frequently associated with oliguria. Hydration therapy remains the cornerstone of prevention. Both short-term and low-volume hydration protocols have been shown to be effective and safe, even in patients treated with intermediate or high doses [202]. Adjunct magnesium administration in such regimens can further reduce nephrotoxicity, although its benefit appears limited at very high cisplatin doses. Mannitol, by inducing osmotic diuresis, is often employed in high-dose regimens, while furosemide use is more controversial due to risks of electrolyte imbalance and secondary hypomagnesemia, which may paradoxically worsen renal injury [203]. Additional support for the protective role of magnesium comes from studies showing that supplementation with 8–16 mEq in short-term, low-volume hydration protocols significantly reduces nephrotoxicity at intermediate cisplatin doses [202]. Alternative agents have been tested with mixed outcomes: glutathione provided a degree of protection comparable to magnesium, whereas dopamine was ineffective. Interestingly, magnesium supplementation seems less effective in patients receiving high-dose cisplatin, where mannitol has demonstrated greater benefit. As an osmotic diuretic, mannitol enhances urine output and thereby reduces tubular cisplatin accumulation; however, it is only recommended in high-dose regimens (≈100 mg/m^2^), since its strong diuretic effect can otherwise lead to dehydration [202]. Replacement of mannitol with furosemide is not well supported by clinical evidence, and carries additional risks [204,205]. By blocking the Na^+^/K^+^/2Cl^−^ cotransporter in the loop of Henle, furosemide may precipitate hypokalemia and secondary hypomagnesemia. This imbalance can further stimulate OCT2 activity, enhancing cisplatin uptake into proximal tubular epithelial cells and paradoxically exacerbating nephrotoxicity [206].

Cisplatin-induced neuropathy manifests primarily as a peripheral sensory neuropathy, with a symmetrical distribution in the extremities that can progress to the knees and elbows [207]. Symptoms include paresthesia, pain, decreased vibratory sensation, mechanical allodynia, and gait disturbances. These deficits can persist or even worsen after therapy cessation, significantly impairing quality of life and sometimes necessitating discontinuation of treatment [208]. In severe cases, degeneration of large myelinated fibers in the dorsal columns may occur, giving rise to Lhermitte’s sign [209]. Effective treatments are lacking: antioxidants such as vitamin E and α-lipoic acid, beneficial in diabetic neuropathy, have not proven successful in this context [210].

Ototoxicity represents another major complication, characterized by bilateral, progressive, high-frequency hearing loss, often accompanied by tinnitus and ear pain [211]. Cisplatin accumulation in cochlear cells is facilitated by CTR1 and OCT2, the same transporters involved in nephrotoxicity. The injury is mediated by oxidative stress and depletion of antioxidant defenses. Pediatric patients appear especially vulnerable, though sodium thiosulfate has shown some protective effect when carefully timed to avoid inactivation of cisplatin’s antitumor activity [212]. Amifostine, another thiol compound, has produced inconsistent results, offering reliable protection against nephrotoxicity, but only partial or inconsistent benefit in reducing ototoxicity or neuropathy [210].

While peripheral neuropathy is the predominant neurotoxic effect, central nervous system complications such as posterior reversible encephalopathy syndrome (PRES) have also been described, particularly in patients receiving cisplatin for brain malignancies. These cases suggest that cisplatin may cross or disrupt the blood–brain barrier, possibly facilitated by pro-inflammatory cytokines and oxidative stress [210]. Symptoms typically reverse after cessation of therapy, though further studies are required to clarify mechanisms.

Beyond the major target organs, other tissues—including the gastrointestinal tract, bone marrow, and reproductive system—are also subject to cisplatin-induced injury, though to a lesser extent. Together, these toxicities underscore the delicate balance between therapeutic efficacy and systemic harm that defines cisplatin-based chemotherapy.

## 7. Conclusions and Perspectives

Cisplatin is a powerful chemotherapeutic agent frequently employed in managing a variety of solid malignancies. It plays a crucial role in treating cancers such as those of the lung, breast, brain, ovary, kidney, testis, and the hematopoietic system. Its primary mechanism involves interfering with cellular DNA, leading to disrupted replication and cell division, ultimately resulting in programmed cell death. Beyond its direct genotoxic effects, cisplatin also triggers oxidative damage through reactive oxygen species generation, promotes cell cycle arrest via p53 activation, suppresses oncogene expression, and activates multiple apoptotic pathways. Despite its clinical success, the use of cisplatin often leads to harmful effects on vital organs, including the liver, kidneys, heart, nervous system, and blood cells. Furthermore, tumor recurrence and resistance to cisplatin remain significant hurdles. To mitigate these challenges, combination regimens incorporating cisplatin and other agents have gained traction, aiming to improve efficacy and minimize toxicity. Emerging evidence supports multi-targeted approaches that not only enhance drug responsiveness but also combat inflammation and limit cellular uptake of cisplatin, paving the way for more effective and safer cancer treatments. In addition to its well-established cytotoxic effects, accumulating evidence suggests that cisplatin can modulate antitumor immunity by promoting forms of immunogenic cell death characterized by the release of danger-associated molecular patterns, enhanced antigen presentation, and activation of dendritic cells [213]. These immunomodulatory effects may contribute to treatment efficacy in selected contexts and provide a biological rationale for combining cisplatin with immune checkpoint inhibitors or other immunotherapies. Looking ahead, major challenges include reducing cisplatin-associated organ toxicity, preventing or reversing drug resistance, and identifying biomarkers that enable patient stratification and personalized dosing strategies. Parallel efforts aimed at developing next-generation platinum compounds with improved selectivity, as well as rational combination regimens that exploit vulnerabilities in DNA repair, redox balance, and immune signaling pathways, are likely to shape the future clinical use of platinum-based chemotherapy.

## Data Availability

No new data were created or analyzed in this study.

## Abbreviations

The following abbreviations are used in this manuscript:

ABC	ATP-binding cassette transporters
AKT/PKB	Protein kinase B
APAF-1	Apoptotic protease-activating factor 1
ATP7A/ATP7B	Copper-transporting ATPases
AQP	Aquaporin
ATM	Ataxia Telangiectasia Mutated
ATR	ATM and Rad3-related
BAD	Bcl-2-associated agonist of cell death
Bax	Bcl-2-associated X protein
Bcl-2/Bcl-xL	B-cell lymphoma proteins (anti-apoptotic family members)
CAT	Catalase
CD133	Cluster of Differentiation 133
CP-r	Cisplatin-resistant
CSCs	cancer stem cells
CTR1	Copper transporter 1
Cyt c	Cytochrome c
DISC	Death-inducing signaling complex
EGFR	Epidermal growth factor receptor
ERK	Extracellular signal-regulated kinase
ESA	Epithelial-specific antigen
FADD	Fas-associated death domain protein
FoxO	Forkhead box O
Fas/FasL	Fas receptor/Fas ligand
GADD45	Growth arrest and DNA damage-inducible 45
GSDM	gasdermin
GLUT1	Glucose transporter 1
GBM	Glioblastoma multiforme
GRP78	Glucose-regulated protein 78
GSCs	glioma stem-like cells
GSH	Glutathione
HER2/ERBB2	Human epidermal growth factor receptor 2
HMG1	High Mobility Group Box 1 protein
HSP	Heat shock proteins (HSP27, HSP60, HSP70, HSP72, HSP90)
JNK	c-Jun N-terminal kinase
MAPK	Mitogen-activated protein kinase
MAST1	Microtubule-associated serine/threonine kinase 1
Mdm2	Mouse double minute 2 homolog
MGMT	O6-methylguanine-DNA methyltransferase
MITF	Microphthalmia-associated transcription factor
MMR	Mismatch Repair
MPT	Mitochondrial permeability transition
MDR1	Multidrug Resistance protein 1/P-glycoprotein
MRP	Multidrug resistance associated proteins
mtDNA	Mitochondrial DNA
NADH	Nicotinamide adenine dinucleotide (reduced form)
NER	Nucleotide excision repair
NF-κB	Nuclear factor kappa-light-chain-enhancer of activated B cells
NMSC	Non-melanoma skin cancer
NOXA	Pro-apoptotic Bcl-2 family protein
Nrf2	Nuclear factor erythroid 2–related factor 2
NSCLC	Non-small-cell lung cancer
OAT	Organic anion transporter
OCT	Organic cation transporter
PARP	Poly(ADP-ribose) polymerase
PI3K	Phosphoinositide 3-kinase
PIDD	p53-induced protein with death domain
PKC	Protein kinase C
POLH/POLI/POLK/REV1/REV3/REV7	Translesion DNA polymerases
PTEN	Phosphatase and tensin homolog
PUMA	p53 upregulated modulator of apoptosis
ROS	Reactive oxygen species
SLC	Solute carrier transporters (SLC22, SLC31 families)
SOD	Superoxide dismutase
S1PR1	Sphingosine-1-phosphate receptor 1
STAT3	Signal transducer and activator of transcription 3
TFAM	Mitochondrial transcription factor A
TMEM205	Transmembrane protein 205
TNF-α	Tumor necrosis factor alpha
5-FU	5-fluorouracil
